# Localized Pantothenic Acid (Vitamin B5) Reductions Present Throughout the Dementia with Lewy Bodies Brain

**DOI:** 10.3233/JPD-240075

**Published:** 2024-07-23

**Authors:** Melissa Scholefield, Stephanie J. Church, Jingshu Xu, Stefano Patassini, Garth J.S. Cooper

**Affiliations:** a Division of Cardiovascular Sciences, School of Medical Sciences, Faculty of Biology, Medicine and Health, The University of Manchester, Manchester, UK; b School of Biological Sciences, Faculty of Science, University of Auckland, Private Bag, Auckland, New Zealand

**Keywords:** Pantothenic acid, vitamin B5, dementia with Lewy bodies, mass spectrometry, metabolomics, UHPLC–MS/MS, Lewy body dementia

## Abstract

**Background:**

Localized pantothenic acid deficiencies have been observed in several neurodegenerative diseases such as Alzheimer’s disease (AD), Parkinson’s disease dementia (PDD), and Huntington’s disease (HD), indicating downstream energetic pathway perturbations. However, no studies have yet been performed to see whether such deficiencies occur across the dementia with Lewy bodies (DLB) brain, or what the pattern of such dysregulation may be.

**Objective:**

Firstly, this study aimed to quantify pantothenic acid levels across ten regions of the brain in order to determine the localization of any pantothenic acid dysregulation in DLB. Secondly, the localization of pantothenic acid alterations was compared to that previously in AD, PDD, and HD brains.

**Methods:**

Pantothenic acid levels were determined in 20 individuals with DLB and 19 controls by ultra-high performance liquid chromatography-tandem mass spectrometry (UHPLC–MS/MS) across ten brain regions. Case–control differences were determined by nonparametric Mann–Whitney U test, with the calculation of S-values, risk ratios, E-values, and effect sizes. The results were compared with those previously obtained in DLB, AD, and HD.

**Results:**

Pantothenic acid levels were significantly decreased in six of the ten investigated brain regions: the pons, substantia nigra, motor cortex, middle temporal gyrus, primary visual cortex, and hippocampus. This level of pantothenic acid dysregulation is most similar to that of the AD brain, in which pantothenic acid is also decreased in the motor cortex, middle temporal gyrus, primary visual cortex, and hippocampus. DLB appears to differ from other neurodegenerative diseases in being the only of the four to not show pantothenic acid dysregulation in the cerebellum.

**Conclusions:**

Pantothenic acid deficiency appears to be a shared mechanism of several neurodegenerative diseases, although differences in the localization of this dysregulation may contribute to the differing clinical pathways observed in these conditions.

## Plain Language Summary

Decreases in a molecule called pantothenic acid (also known as vitamin B5) have been observed in several areas of the brain in multiple dementia disease, including Alzheimer’s disease, Parkinson’s disease dementia, and Huntington’s disease. However, it is unknown whether such changes also occur in another dementia disease, dementia with Lewy bodies, which shows many of the same symptoms and molecular changes as these conditions. As such, this study was performed in order to determine if and where changes in pantothenic acid occur throughout the dementia with Lewy bodies brain. Using a methodology called liquid chromatography–mass spectrometry, which is able to measure pantothenic acid levels in a highly precise manner in brain tissues, we found that several regions of the dementia with Lewy bodies brain show decreases in pantothenic acid, including some involved in movement such as the substantia nigra and motor cortex, as well as regions associated with cognition and memory such as the hippocampus—looking most similar to the pattern of changes already seen in Alzheimer’s disease. It is possible that these changes contribute to the progression of dementia with Lewy bodies; however, further studies need to be performed to determine at what point these changes happen during the disease and how they may contribute to the development of symptoms.

## INTRODUCTION

Dementia with Lewy bodies (DLB) is a neurodegenerative disease that is clinically diagnosed by the presence of Parkinsonian motor dysfunction presenting one year or more following the onset of cognitive decline. This is in contrast to Parkinson’s disease dementia (PDD), in which motor dysfunction precedes cognitive decline [1]. At present, the relative onset of cognitive and motor dysfunction is the primary means of distinguishing DLB from PDD, as otherwise their clinical and neuropathological presentation can be very similar. Neuropathologically, both conditions present with extensive dopaminergic neuronal loss, primarily within the substantia nigra pars compacta (SNpc, but spreading throughout the brain as the disease progresses. This neuronal loss is accompanied by extensive deposition of *α*-synuclein-containing Lewy bodies. Although DLB may present with a somewhat more cortical deposition of Lewy bodies [2], this is not always the case, and the end-stage neuropathological presentation of these two diseases makes them difficult to distinguish even at postmortem.

Further complicating matters, DLB also shows many similarities to another common neurodegenerative disease—Alzheimer’s disease (AD). Prior to the onset of motor dysfunction, DLB can be very difficult to distinguish from AD in the clinic. Supporting clinical criteria include the presence of REM-behavior sleep disorder (RBD), restless legs syndrome, and hallucinations [1], but these may not always be present. Neuropathologically, AD is characterized primarily by cholinergic neuronal loss within the hippocampus (HP), accompanies by misfolded amyloid-*β* and tau inclusions. However, as up to two thirds of DLB cases may present with amyloid-*β* and tau pathology at postmortem [3], and up to half of AD cases can show *α*-synuclein pathology [4], definitive diagnosis remains a challenge. It has even been suggested that up to half of dementia cases may in fact be cases of mixed dementia [5], further complicating diagnosis. This can cause issues in admitting DLB patients to the correct treatment pathway, with some therapeutics that are safe to use in cases of PDD and AD—such as typical or traditional antipsychotics—being harmful if administered to individuals with DLB [6].

These issues have raised the importance of identifying additional clinical or neuropathological criteria by which we may be able to distinguish DLB from other similar neurodegenerative conditions, as well as identifying the mechanistic pathways that are involved in both the differing clinical presentation of DLB and the necessity of differing therapeutic pathways for its treatment. One such possible criterion may be the presence of localized pantothenic acid (AKA vitamin B5) deficiencies across the brains of individuals with different neurodegenerative diseases. Pantothenic acid is an essential vitamin for the human body, essential for the synthesis of coenzyme A (CoA)—a molecule that plays essential vital roles in many metabolic processes including neurotransmitter synthesis, fatty acid metabolism, and bioenergetic pathways such as the citric acid cycle [7]. Previous studies performed by our lab using directly comparable methodologies have identified localized decreases in the SN, medulla (MED), and pons of the PDD brain [8], in the motor cortex (MCX), primary visual cortex (PVC), HP, middle temporal gyrus (MTG), and cingulate gyrus (CG) of the AD brain [9], as well as in the HP, SN, putamen (PUT), globus pallidus (GP), middle frontal gyrus (MFG), and entorhinal cortex (ENT) of the Huntington’s disease (HD) brain [10]—with the cerebellum (CB) being the only region observed to be altered across all three conditions. Such dysregulations in pantothenic acid levels may indicate the presence of localized deficiencies in several downstream pathways, in particular energy production pathways, contributing to the pathogenesis of these diseases.

The aim of this study was to carry out a similar investigation across multiple regions of the DLB brain, using these methods in order to identify whether pantothenic acid alterations are present in the DLB brain, where such changes are localized, and whether the pattern of the pantothenic alterations in the DLB brain is similar or different to that observed in AD, PDD, and HD. This study represents the first such investigation of pantothenic acid levels in the DLB brain.

## METHODS

The studies involving human participants were reviewed and approved by Manchester REC (09/H0906/52 + 5), NZ Neurological Foundation Douglas Human Brain Bank, and the UK MRC Brain Bank Network. The patients/participants provided their written informed consent to participate in this study. The high values of many of the additional statistical tests performed, such as the risk ratios, E-values, S-values, and effect sizes, also indicate the reliability of the current study’s findings.

### Acquiring tissue for pantothenic acid quantification

For the purposes of this study, brain tissue from ten regions including the cerebellum at the level of the dentate nucleus (CB), motor cortex (MCX), primary visual cortex (PVC), hippocampus (HP), pons, anterior cingulate gyrus (CG), medulla (MED), substantia nigra (SN), putamen (PUT), and middle temporal gyrus (MTG) were obtained from 20 confirmed cases of DLB and 19 dementia-free controls from the Sepulveda and Harvard brain banks under the NIH NeuroBioBank network. Brain regions were selected based on two criteria: 1) Coverage of not only regions with typically high levels of neurodegeneration, but also those with moderate and low neuronal loss and protein deposition, in order to ascertain whether pantothenic acid alterations match the typical pattern of neurodegeneration in DLB and 2) Coverage of regions that have been previously assessed in AD, PDD, and HD in order to be able to perform direct regional comparisons.

All patient data available from the brain banks was collected and recorded, including age at death, sex, postmortem delay (PMD), *α*-synuclein Braak stage, DLB subtype, and comorbidities (see Supplementary Material A for individual patient data). Cases and controls were matched as closely as possible across these criteria for each individual region. Where possible, PMD was kept below 24 hours in accordance with a previous study conducted by our lab in which pantothenic acid levels were not found to change in the rat cortex or cerebellum within a period of up to 24 hours [11].

### Diagnosis of DLB cases

Cases and controls were diagnosed by the referring neuropathologists of the Sepulveda and Harvard brain banks under the NIH NeuroBioBank framework. DLB cases had all received a clinical diagnosis of DLB during their lifetime according to the DLB Consortium Criteria [1] and a neuropathological diagnosis of Lewy body dementia (LBD) according to the McKeith criteria [1]; rather than being staged according to the *α*-synuclein Braak stage, DLB cases were subtyped as brainstem-predominant, amygdala-predominant, olfactory bulb only limbic, or diffuse/cortical DLB, according to previously defined criteria [1]. In short, these subtypes are defined by according to the distribution of a-synuclein pathology throughout the brain, which does not always follow the pattern of traditional Braak staging [12]. Mixed dementia DLB/AD cases were also identified according to the presence of AD neuropathology [13], with Braak tau scores included where possible. Controls were not diagnosed with any form of dementia during their lifetime, showed no *α*-synuclein pathology, and had tau Braak scores of <2.

### Pantothenic acid quantification

Pantothenic acid quantification was performed using previously validated methods [14]. In short, Samples were extracted into 50 : 50 (v/v) methanol:chloroform containing known concentrations of heavy-labelled pantothenic acid standard (Vitamin B5 (di-*β*-alanine-^13^C_6_,^15^N_2_); Sigma-Aldrich, Missouri, USA), used to correct for intra- and inter-run variability. Methanol:chloroform:internal standard blanks were also prepared. Samples were lysed in a TissueLyser batch bead homogeniser (Qiagen, Manchester, UK) using carbamide beads. LC–MS grade water was added to lysed samples before centrifugation at 2400  ×g to separate polar and non-polar phases. The polar methanol phase was transferred to a fresh tube before drying overnight in a Speedvac centrifugal concentrator (Savant Speedvac, Thermo Scientific, UK).

Following drying, 0.1% formic acid was added to each sample and blank. This solution was then transferred to 300*μ*l autosampler vials (Thermo Fisher Scientific, Massachusetts, USA). Four blanks containing only 0.1% formic acid were also prepared and interleaved throughout the UHPLC–MS/MS run. Standard solutions containing the heavy-labelled pantothenic acid internal standard (Vitamin B5 (di-*β*-alanine-^13^C_6_,^15^N_2_) calcium salt ≥98 atom%, ≥97% (CP), 705837 Sigma-Aldrich) and unlabelled pantothenic acid external standards (D-Pantothenic acid hemicalcium salt ≥98.0%, 1210 Sigma-Aldrich) in 0.1% formic acid were prepared in 300*μ*l autosampler vials; containing concentrations of 0–5000 nM unlabelled and 500 nM labelled pantothenic acid. These were used to create standard curves during analysis, which were used to determine the concentration of pantothenic acid within samples. Three QCs containing 20, 200, and 2000 nM of unlabelled and 500 nM labelled pantothenic acid standards in 0.1% formic acid were also prepared and interleaved throughout the run to account for intra-run variability.

Pantothenic acid quantification was performed on an Accela UHPLC–MS (Thermo Fisher Scientific) using a Hypersil Gold AQ column with a diameter of 2.1 mm, length of 100 mm, and particle size of 1.9*μ*m in reverse phase mode (Thermo Fisher Scientific 25302-101130) and 0.5*μ*m pre-column filter (Thermo Fisher Scientific 22016). The column was maintained at 25°C with a nitrogen carrier gas at a flow rate of 300*μ*l/min. An electrospray ionization source was used with argon collision gas. Two regions were analyzed per run, with randomization of cases and controls; inter-run and intra-run variability was controlled for with the addition of known concentrations of spiked internal standards.

### UHPLC-MS/MS data analysis

Peaks were identified based on the expected retention time (RT) based on comparison with the RT of the spiked pantothenic acid labelled internal standard. Peaks were manually checked to ensure correct identification by the software. Standards were only accepted when showing a % difference of <15%, with 6/10 standards required for acceptance of the standard curve. QCs showing a % difference >20% were excluded, with 2/3 successful QCs required in each set for the run to be accepted.

Concentrations of pantothenic acid in each sample was determined based on the standard curve. Concentrations were corrected for sample wet-weight and case-control differences analyzed in GraphPad Prism v8.1.2. (Prism; La Jolla, CA). A non-parametric Mann–Whitney U test was used due to the small sample size. A *p*-value < 0.05 was considered significant. Shannon diversity indices (S-values AKA surprisal scores) were also calculated by taking the negative base 2 log of the *p*-value. Confidence intervals were calculated using the following equation:

(1) CI=SE*Z(0.95)

where SE = the standard error and Z(0.95) = the z-score corresponding to a confidence level of 0.95. Mann–Whitney U calculations were performed using GraphPad v8.1.2 (Prism; La Jolla, CA). *p*-values < 0.05 were considered significant.

In order to determine the statistical power of the data obtained, a *post hoc* power analysis was performed. The statistical power was determined for each region at both *p* < 0.05 and *p* < 0.01 using the ClinCalc Post Hoc Power Calculator (ClinCalc LLC., Chicago, IL, USA) available at https://clincalc.com/Stats/Power.aspx. A statistical power of >80% was considered good. In addition to this, the minimum number of samples required to confidently determine case–control differences at a significance level of *p* < 0.05 and *p* < 0.01 was calculated using the ClinCalc Sample Size Calculator (ClinCalc LLC., Chicago, IL, USA) available at https://clincalc.com/Stats/SampleSize.aspx.

### Sensitivity analyses

In order to assess whether the interpretation of the data obtained in the current study was appropriate and robust, a sensitivity analysis was performed for every significant (*p* < 0.05) case–control difference in metal levels. For both individual runs and the mean values of all three replicate runs taken together, the risk ratio (RR), E-value, and effect size were determined. An explanation of these is given below.

The risk ratio is used to compare the risk of a ‘health event’ between different groups (in our case, to compare DLB cases and controls); it is determined by the following equation:

(2) $$\mathrm{RR}=\left(\frac{a}{b}\right)/-\left(\frac{c}{d}\right)$$

Where ***a*** = the number of case values >95% upper CI limit of the controls (or <95% lower CI limit where significant *decreases* were observed in cases), ***b*** = number of cases, ***c*** = number of control values >95% upper CI limit of the controls (or <95% lower CI limit where significant *decreases* were observed in cases), and ***d*** = number of controls. Risk ratios of >3 were considered to be robust. In the case of null values in the calculation of risk ratios, the null values were assigned a value of 0.5.

E-values were calculated for risk ratios as well as for the upper and low confidence limits of the risk ratios. The E-value defines the minimum strength of association, on the risk ratio scale, that a potential confounder would have to have with both a treatment (e.g., urea levels) and an outcome (e.g., an increased risk of DLB) to explain away an observed treatment–outcome association (i.e., the observed association between concentrations of urea and an increased risk of DLB), while taking into account measured covariates (here including age, sex, and PMD). The higher the E-value, the stronger the confounding required to nullify the treatment–outcome association. The E-value was calculated using Equation (3):

(3) E value=RR+sqrt(RR x (RR–1))

In the calculation of E-values for RR < 1, the inverse of the risk ratio was first taken. E-values were also calculated for the confidence intervals of the risk ratios; if the range of the confidence intervals crossed 1.0, then the E-value was determined to be 1.0; otherwise, E-values were calculated according to Equation 5, substituting the RR for the CI closest to 1.0.

The effect size describes the *strength* of the relationship observed between variables, rather than indicating whether differences are present due to chance or otherwise. Determination of effect sizes can indicate where significantly altered (*p* < 0.05) variables have a negligible influence on an outcome, or conversely where variables found to be non-significant (*p* < 0.05) in traditional statistical testing have a large contribution towards an outcome; the latter may occur where statistical power is low due to small sample sizes. The effect size was here determined using Glass’ Delta:

(4) $${\mathrm{Glass}}^{\prime }\Delta =(M_{1}-M_{2})/\sigma \;\mathrm{control}$$

where M_1_ = mean case value, M_2_ = mean control value, and *σ* control = standard deviation of the control group; M_1_ and M_2_ were reversed in case of significant decreases. Glass’ delta was used as the group sample sizes were equal, but their standard deviations were unequal. Effect size values 0.2–0.5 were considered small, values between 0.50 and 0.80 were considered of medium size, values between 0.80 and 1.30 were considered large, and effect sizes >1.30 were considered very large.

## RESULTS

### Cohort characteristics

For this study, brain tissue was obtained from 10 regions from a total of 20 DLB cases and 19 controls. As not every donor had tissue available for every investigated brain region, individual n numbers varied per region (see Table 1).

**Table 1:** Number of cases and controls per investigated region

	HP	MED	MTG	CG	PVC	PONS	MCX	PUT	SN	CB
Controls	14	15	15	15	16	14	16	13	18	16
Cases	15	15	15	15	15	8	15	8	15	12

Cases and controls were matched as closely as possible for sex, age at death, and PMD. There were no significant differences in the overall cohort or any individual brain region for sex or age at death (See Table 2). For PMD, there was a slightly shorter PMD in cases than controls in the overall cohort (see Supplementary Table A2). In accordance with a previous study conducted by our group assessing the effects of PMD on various metabolites in the brain, the PMD was kept as close to 24 hours or below as possible, as pantothenic acid levels were not found to alter within this period of time [11].

**Table 2:** Overall cohort characteristics

	Age at death (y)	Sex (% Male)	PMD (h)
Controls (*n* = 19)	73.6 (65–85)	57.9	18.4 (8.1–29.1)
Cases (*n* = 20)	74.1 (65–85)	60.0	14.7 (8.0–22.6)^*^

Table shows data for age and PMD as mean (range). PMD, postmortem delay. ^*^p < 0.05.

### Pantothenic acid analysis

**Fig. 1:**
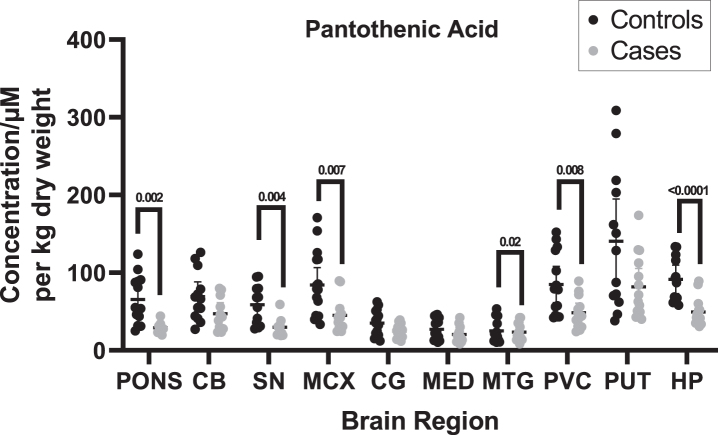
**Pantothenic acid concentrations in DLB cases and controls.** Figure shows mean urea concentrations±95% confidence intervals in *μ*M/Kg. Case–control differences were determined using Mann–Whitney U test. CB, Cerebellum; SN, Substantia nigra; MCX, Motor cortex; CG, Cingulate gyrus; MED, Medulla; MTG, Middle temporal gyrus; PVC, Primary visual cortex; PUT, Putamen; HP, Hippocampus.

Pantothenic acid concentrations were measured in DLB cases and controls across ten regions of the brain using UHPLC-MS/MS. Of the ten investigated regions, six showed significant (*p* < 0.05) decreases in cases compared to controls (See Table 3 and Fig. 1; for individual values, see Supplementary Material B), including the pons (29.3 vs. 32.6*μ*M/kg; *p* = 0.002), SN (47.1 vs. 58.9*μ*M/kg; *p* = 0.004), MCX (45.0 vs. 84.1*μ*M/kg; *p* = 0.007), MTG (29.7 vs. 47.1*μ*M/kg; *p* = 0.02), PVC (48.6 vs. 91.5*μ*M/kg; *p* = 0.008), and HP (49.4 vs. 91.5*μ*M/kg; *p* < 0.0001) as determined by non-parametric Mann–Whitney U tests. No statistically significant differences were found in the CB, CG, MED, or PUT.

**Table 3:** Pantothenic acid concentrations in DLB cases and controls

Region	Controls (*μ*M/kg)	Cases (*μ*M/kg)	Fold-change	*p*	S-value	E-value	RR	RR CI E-value	Effect Size
PONS	32.6±15.4 (19.7–45.4)	29.3±7.5 (23.0–35.5)	0.9	0.002	9.3	8.8	4.7 (1.7–12.7)	2.8	–1.2
CB	69.1±31.7 (49.9–88.3)	47.1±21.3 (32.8–61.4)	0.7	0.1	3.1	2.9	1.8 (0.7–4.7)	1.0	–0.7
SN	58.9±25.5 (42.7–75.0)	29.7±11.8 (21.8–37.6)	0.5	0.004	8.0	4.9	2.7 (1.2–6.2)	1.7	–1.2
MCX	84.1±42.0 (61.7–106.5)	45.0±21.5 (32.1–58.0)	0.5	0.007	8.1	4.9	2.7 (1.3–5.8)	1.8	–0.9
CG	35.3±16.2 (26.3–44.3)	25.2±8.6 (20.2–30.2)	0.7	0.09	4.5	2.2	1.4 (0.7–3.1)	1.0	–0.6
MTG	47.1±22.9 (34.5–59.8)	29.7±18.9 (13.9–24.3)	0.6	0.02	6.0	3.4	2.0 (0.9–4.5)	1.0	–0.8
MED	27.3±13.7 (19.4–35.2)	20.4±10.1 (14.5–26.2)	0.8	0.2	2.3	1.6	1.2 (0.5–2.7)	1.0	–0.5
PVC	91.5±46.5 (65.7–117.2)	48.6±20.2 (36.4–60.8)	0.5	0.008	7.9	3.3	1.9 (0.97–3.8)	1.0	–0.9
PUT	140.6±89.5 (86.5–194.7)	81.8±40.9 (58.2–105.3)	0.6	0.09	4.5	3.1	1.9 (0.7–4.7)	1.0	–0.7
HP	91.5±28.7 (73.3–109.8)	49.4±17.8 (39.1–59.7)	0.5	<0.0001	11.5	3.5	2.1 (1.02–4.2)	1.2	–1.5

Table shows mean pantothenic acid concentration with 95% confidence intervals in *μ*M/kg. Case–control differences were determined using Mann–Whitney U tests. *p* < 0.05 was considered significant. Fold-changes are cases compared to controls. See Supplementary Material B for individual data. RR, Risk ratio.

When determining whether pantothenic acid level changes are present in different brain regions, other measures as well as statistical significance were considered, such as the effect size and E-values. The effect sizes ranged from medium to very large, even in regions where statistically significant differences were not observed based on *p*-values, ranging from –0.5 in the MED to as high as –1.5 in the HP. In statistically significant regions, all effect sizes were large (0.8–1.3) or very large (>1.3). E-values ranged from 1.6 in the MED to 8.8 in the pons; the lowest E-value for a statistically significant region was 3.3 in the PVC. Risk ratios ranged from 1.4 in the CG to 4.7 in the pons; for statistically significant regions, risk ratios ranged from 1.9 in the PVC to 4.7 in the pons, only reaching a robust value of >3 in the pons.

A post hoc power analysis was performed for each investigated brain region to determine the statistical power of the obtained data (see Supplementary Table A10). A statistical power of ≥80% was considered good. All regions showing *p* < 0.05 had a statistical power of ≥80%, with the HP showing the highest power at 99.3%, followed by the pons at 98.9%, the SN at 94.6%, and the MCX and PVC both at 89.9%.

### Comparison to PDD, AD, and HD

**Fig. 2:**
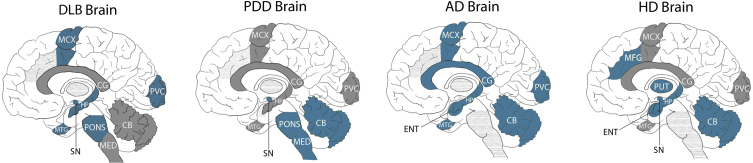
**Comparison of pantothenic acid alterations in DLB, PDD, AD, and HD brains.** Blue regions indicate decreases in pantothenic acid. Grey regions indicate no changes in pantothenic acid. Grey dotted shading indicates that region was not investigated in that condition. CB, Cerebellum; CG, Cingulate Gyrus; ENT, Entorhinal Cortex; HP, Hippocampus; MED, Medulla; MCX, Motor Cortex; MFG, Middle frontal gyrus; MTG, Middle Temporal Gyrus; PUT, Putamen; PVC, Primary visual cortex; SN, Substantia Nigra.

Pantothenic acid levels have also been measured by our lab in PDD, AD, and HD brains using the same methodologies as employed in the current study. As such, the results of each of these investigations can be compared directly across several regions of the brain (see Table 4 and Fig. 2). For DLB, results can be directly compared with nine regions of the PDD brain (CB, MCX, PVC, HP, SN, MTG, MED, CG, and pons; see Table 4), six regions of the AD brain (CB, MCX, PVC, HP, MTG, and CG), and seven regions of the HD brain (CB, MCX, PVC, HP, SN, MTG, and CG). Results are comparable across all six conditions for six regions of the brain (CB, MCX, PVC, HP, MTG, and CG). The characteristics of the AD, PDD, and HD cohorts can be found in Supplementary Tables A3–9.

**Table 4:** Pantothenic acid fold-changes in PDD, AD, and HD

Region	Fold-change DLB	Fold-change PDD [8]	Fold-change AD [9]	Fold-change HD [10]
CB	0.7	**0.6**	**0.5**	**0.6**
MCX	**0.5**	0.8	**0.3**	0.6
PVC	**0.5**	0.9	**0.4**	0.5
HP	**0.5**	0.9	**0.5**	**0.5**
SN	**0.5**	**0.6**	–	**0.6**
MTG	**0.6**	0.9	**0.5**	0.6
MED	0.7	**0.6**	–	**–**
CG	0.7	0.8	**0.5**	0.5
PONS	**0.9**	*0.7*	–	–
PUT	0.6	–	–	**0.5**
GP	–	–	–	**0.4**
MFG	–	–	–	**0.6**
ENT	–	–	**0.4**	**0.6**

Table shows case-control fold-changes in urea. CB, cerebellum; MCX, motor cortex; PVC, primary visual cortex; HP, hippocampus; SN, substantia nigra; MTG, middle temporal gyrus; MED, medulla; CG, cingulate gyrus; PUT, putamen; GP, globus pallidus; MFG, middle frontal gyrus; ENT, entorhinal cortex. Significant intra-cohort case–control fold-changes are highlighted in bold.

DLB is the first of these diseases to not show significant case–control differences in pantothenic acid in the CB, indicating that the relative protection of this region may be useful for distinguishing DLB from other neurodegenerative diseases. In terms of shared alterations, DLB looks the most similar to AD, with both showing significant decreases in pantothenic acid in four regions: the MCX, PVC, HP, and MTG. This result reinforces the neuropathological similarities already observed between these two conditions.

The extent of pantothenic acid deficiency in affected regions appears to be similar across both regions and diseases, with an average fold-change of 0.5–0.6 across all four diseases, with few regions showing more (0.3 in the AD MCX) or less (0.9 in the DLB pons) extensive decreases.

DLB, PDD, and HD all show significant decreases in the SN, indicating the vulnerability of this region in neurodegenerative diseases associated with motor dysfunction; as this region was not investigated in AD, it is unknown whether this region is spared in dementia diseases that are not associated with motor symptoms. DLB, HD, and AD all show pantothenic acid decreases in the HP, but this region appears to be shared in the PDD, which may be associated with differing levels of cognitive decline in these conditions. DLB shows no further shared alterations with either HD or PDD, despite its extensive clinical and neuropathological similarities with the latter.

## DISCUSSION

This study represents the first multi-regional investigation of pantothenic levels across the DLB brain, observing statistically significant decreases across six of the ten investigated regions. The pattern of pantothenic acid alterations appears most similar to that already observed in AD, with few similarities to PDD and HD. The regions affected include those with the highest typical levels of neurodegeneration and Lewy body deposition in DLB, such as the SN, HP, and MCX, with relatively spared regions such as the CB, MED, and PUT showing no alterations. Unlike other neurodegenerative diseases, the CB appears to be protected from pantothenic acid alterations in DLB, although decreases in the substantia nigra appear to be a common observation in conditions associated with motor dysfunction (DLB, PDD, HD).

### Pantothenic acid

Pantothenic acid is an essential B vitamin that acts as the precursor to CoA, a vital component of a vast number of metabolic pathways including fatty acid synthesis (and subsequent regulation of excess glucose levels); energy production via the citric acid cycle; antioxidation; lipid, carbohydrate, and amino acid metabolism; protein synthesis and post-translation protein modification; and many others [7]. Overall, CoA serves as a cofactor for around 4% of known enzymes—as such, any alterations in CoA levels could have a multitude of downstream metabolic effects. Such effects have been observed in the AD brain, with widespread perturbations in several CoA-modulated enzymes and enzyme complexes—pyruvate dehydrogenase complex, isocitrate dehydrogenase, 2-oxoglutarate dehydrogenase complex, and succinyl-CoA synthetase—being reported [15]. CoA’s roles in protein modification and antioxidation may play a key role in tauopathies such as AD and DLB, with the CoAlation of tau protein in AD brain samples being observed to protect against the H_2_O_2_-induced dimerization of tau [16]; a loss of CoA as a result of pantothenic acid deficiency may lead to the loss of this protective mechanism. Indeed, increased levels of CoA have been suggested to protect against general cognitive decline in the aging brain [17].

Pantothenic acid is generally obtained from the diet, being available from a wide variety of food sources and occurring in particularly high levels in eggs, beef, chicken, some vegetables, and fortified cereals [18]. Due to the ease of obtaining sufficient amounts of pantothenic acid from food sources (5 mg daily for adults [19]), deficiency is rare, and usually only present in individuals with severe general malnutrition. However, mutations in the pantothenate kinase 2 (PANK2) gene on chromosome 20 may result in pantothenic acid deficiency, with downstream CoA deficiency [20]. These mutations can result in a condition called pantothenate-kinase associated neurodegeneration (PKAN), a condition that arises in early life characterized by motor dysfunction that eventually leads to issues with falls, chewing, and swallowing [20]. Motor dysfunction may appear very similar to that seen in PD/DLB, and psychiatric disturbances may also occur. Cerebral iron accumulation also occurs in PKAN, as has also been observed in some studies of the PD/PDD brain [21]. There have been some reports of beneficial effects with pantothenic acid supplementation in PKAN, but this is not currently part of the standard treatment pathway for this disease [22].

### Pantothenic acid in neurodegenerative disease

Despite the rarity of pantothenic acid deficiency, significantly lower levels of dietary pantothenic acid intake have been observed in individuals with PD in comparison to healthy individuals, with pantothenic acid levels displaying clinical importance in predicting the incidence of PD [23]; in this study, UPDRS scores were also negatively correlated with pantothenic acid intake. As such, decreased dietary pantothenic acid intake may be associated with both PD incidence and severity. Pantothenic acid intake has also been associated with amyloid-*β* burden in individuals with mild cognitive impairment (MCI), indicating a potential link with AD [24]. As such, despite a lack of outright pantothenic acid deficiency, supplementation may be a potential therapeutic option for the treatment of these neurodegenerative diseases.

To the authors’ knowledge, there have few investigations of absolute cerebral pantothenic acid levels in neurodegenerative diseases other than our own. However, one study has reported decreased levels in the frontal cortex of individuals with AD, identifying pantothenate and CoA biosynthesis as an altered pathway in the AD brain [25]. Conversely, in individuals with MCI, cerebral pantothenic acid levels appear to be positively correlated with amyloid-*β* deposition in the brain [6], with no associations observed with any other investigated vitamin. Individual regional analyses found this association in the frontal, parietal, and temporal cortices, although the relationship did not reach significance in the cingulate gyrus; the discrepancy between these findings and those of our own analysis of the AD brain may reflect regional differences in pantothenic acid dysregulation, as the frontal, parietal, and temporal cortices were not investigated in our study. However, these results do conflict with those of Paglia et al. in the frontal cortex, mentioned above [25]. Furthermore, a study by Čuperlović-Culf et al. observed metabolic network alterations in the neocortex of DLB patients involving pantothenate, despite observing no significant differences in pantothenate concentration between DLB cases and controls [26], giving support for a role of pantothenic acid in the DLB brain.

Non-CNS disturbances in pantothenic acid and related pathways have also been observed in neurodegenerative diseases; for instance, pantothenate and CoA biosynthesis has been found to be disturbed in LC–MS metabolomics analyses of PD and healthy plasma and serum samples, with decreased pantothenic acid levels observed even in the early stages of PD [27, 28]. The presence of pantothenic alterations in the PD gut is disputed, with some studies showing no changes in stool sample levels [29] while others have shown decreases [30], and with another study showing positive associations between fecal pantothenic acid levels and non-motor symptoms in PD [31]. Both increased and decreased serum pantothenic acid levels were reported in a study of 50 individuals with non-specific dementia [32], and pantothenate and CoA biosynthesis pathway dysregulation has been reported in a multi-omic investigation of the AD brain, blood, and cerebrospinal fluid [33]. As such, pantothenic acid disturbances may be a wider, multi-system issue that does not only affect the brain in neurodegenerative diseases; if so, this could make the administration of pantothenic acid supplementation a more straightforward and viable option for therapeutic trials.

### Localization of pantothenic acid deficiency

In the current study, pantothenic acid was observed to be decreased in six regions: the pons, SN, MCX, MTG, PVC, and HP. These regions can be broadly divided into three functional groups, with some overlap between groups—those associated with cognitive function (HP, MTG), those associated with motor function (SN, MCX, PONS), and those associated with visual processing (MTG, PVC). This suggests that pantothenic acid may play a mechanistic role in several aspects of DLB, including the cognitive decline, motor dysfunction, and high prevalence of visual hallucinations observed in this disease.

Conversely, in PDD, the affected regions are primarily those involved in motor dysfunction, including the SN, MED, and pons. As such, the differing localization of pantothenic acid deficiency may account for the difference in the prevalence and onset of motor and cognitive symptoms between DLB and PDD. In AD, the majority of affected regions are those associated with cognitive function, such as the HP, MTG, ENT, and CG, although the MCX and PVC are also affected; the SN, MED, and pons have not been investigated in AD and so the presence of pantothenic acid dysregulation in these regions remains unknown. In AD, every region that was investigated was found to be affected, which may reflect the overall atrophy observed in late-stage AD. In HD, regions involved in both cognitive and motor dysfunction were affected, with a sparing of the PVC. This again suggests that differences in pantothenic acid dysregulation localization may reflect the differing mechanistic processes occurring in these neurodegenerative diseases.

Most strikingly, unlike in PDD, AD, and HD, the cerebellum did not show changes in pantothenic acid levels in DLB. This puts the CB forward as a particularly key brain region in the differentiation of DLB from other neurodegenerative diseases; together with the lack of changes seen in the PDD PVC (in which hallucinations are less likely to occur than in DLB), pantothenic acid levels may aid in the definitive postmortem diagnosis of DLB. Future studies of these conditions should prioritize these brain regions when investigating the mechanistic pathways contributing to the differing clinical progression of these conditions.

### Strengths and limitations

The current study has several strengths and limitations that should be considered when discussing the potential implications of the data obtained. In terms of its strengths, this is the first study, to the authors’ knowledge, in which a multi-regional, fully quantitative study of pantothenic acid levels has been performed in the DLB. It has been carried out using a validated methodology that has also been used to quantify pantothenic acid levels in AD, PDD, and HD, allowing for a direct region-to-region comparison. To improve upon this methodology, a larger cohort was obtained—15 vs. 15 rather than the previous sample sizes of 9 vs. 9—in order to improve confidence in the results obtained. As with many brain tissue studies however, this sample size is still fairly low, and this should be considered when assessing the study’s findings; in particular, regions in which the observed effect sizes were large without reaching statistical significance would benefit from further investigation in larger cohorts.

This study also has limitations; as this is a postmortem tissue study, it is not possible to determine on the basis of this data whether pantothenic acid changes are a cause or a result of pathological changes in DLB. As pantothenic acid levels cannot be determined *in vivo* in the brain with current methods, investigations of peripheral levels, such as in the CSF or plasma, would be necessary to determine the temporality of alterations; this, however, would only be possible in the event that cerebral pantothenic acid changes are reflected in the periphery. There is some evidence that this may be the case, as has been observed in some studies on PD plasma and serum [27, 28]; however, this has yet to be determined in DLB. As such, assessing peripheral pantothenic acid levels in a longitudinal cohort would be an intuitive and informative investigation to follow from the current study.

Another limitation is the lack of some additional data for the cohort used in this study; information such as the *α*-synuclein Braak stage, the duration of disease, and other additional factors would have been useful in the evaluation of the study data. Unfortunately, this data was not available from the brain bank—all data available upon request to the supplying brain banks is available in Supplementary Material A. This also prevented the evaluation of pantothenic acid levels in relation to *α*-synuclein Braak stage; it would be informative to repeat this study in a larger cohort with representation of all *α*-synuclein Braak stages in order to determine any correlation they may show with cerebral pantothenic acid levels.

### Conclusions

Pantothenic acid deficiency appears to be a shared perturbation in the brains of those with DLB, AD, PDD, and HD, despite their clinical and neuropathological differences. Such differences may be accounted for by differences in the localization of pantothenic acid alterations, with regions of the brain associated with motor and cognitive function as well as visual processing affected in the DLB brain. Pantothenic acid alterations should be further investigated for their potential mechanistic role in these diseases by looking at temporal alterations in peripheral pantothenic acid levels. Closer attention should be paid to regions of the brain—such as the PVC and CB—that appear to be able to better differentiate DLB from other, similar neurodegenerative diseases in future studies of the DLB brain.

## AUTHOR CONTRIBUTIONS

MS: Conceptualization, data curation, formal analysis, investigation, methodology, project administration, validation, visualization, writing—original draft, review, and editing. JX, SC, SaPh, and StPa: Investigation and methodology. GJSC: Conceptualization, funding acquisition, resources, supervision, writing—review and editing. MS and GJSC verified the underlying data for this manuscript. All authors read and approved the final version of the manuscript.

## Supplementary Material

Supplementary Material A

Supplementary Material B

## Data Availability

All raw data for individual runs are included in Supplementary Material B; concentrations are given in mm/Kg. Values are given for mean averages, standard deviations, confidence intervals, Welch’s t-test, and Mann-Whitney U test results. Wet weight sample weights are given in mg. Details of all software used for analyses is included in the methods section.
