# Supplementary material for: Localized Pantothenic Acid (Vitamin B5) Reductions Present Throughout the Dementia with Lewy Bodies Brain
Source: J Parkinsons Dis. 2024 Jul 23;14(5):965–76. doi: 10.3233/JPD-240075 (PMC11307062; doi:10.3233/JPD-240075)
Supplement: Supplementary Material A [file jpd-14-jpd240075-s001.pdf]

Supplementary Material A

Localized Pantothenic Acid (Vitamin B5) Reductions Present Throughout the Dementia with Lewy Bodies Brain

Supplementary Table 1. DLB cohort characteristics

ID	Source	Type	Age	Sex	PMD	Race	Clinical Brain Diagnosis	DLB Type	α-syn Braak stage	tau Braak stage	Comorbidities
**C1**	Sepulveda	C	79	Female	14	White	No Dementia	N/A	0	0	Coronary Artery Disease, hypertension
**C2**	Sepulveda	C	72	Male	12.2	White	No Dementia	N/A	0	1	COPD, Pulmonary Emphysema
**C3**	Sepulveda	C	65	Female	17.3	White	No Dementia	N/A	0	0	None reported
**C4**	Harvard	C	69	Female	21.3	Unknown	No Dementia	N/A	0	1	Mild atherosclerosis and arteriosclerosis
**C5**	Harvard	C	66	Male	20.9	White	No Dementia	N/A	0	1	Argyrophilic grain disease (mild), mild arteriosclerosis
**C6**	Harvard	C	74	Female	16.6	Unknown	No Dementia	N/A	0	2	Reactive gliosis
**C7**	Harvard	C	74	Male	14.3	Unknown	No Dementia	N/A	0	2	CVD, atherosclerosis, arteriosclerosis
**C8**	Harvard	C	73	Male	18.0	White	No Dementia	N/A	0	1	Atherosclerosis, arteriosclerosis
**C9**	Harvard	C	68	Male	19.2	Unknown	No Dementia	N/A	0	2	SVD, atherosclerosis, arteriosclerosis, small infarct in pons, three microinfarcts in frontal lobe white matter
**C10**	Harvard	C	66	Male	19.4	White	No Dementia	N/A	0	1	Arteriosclerosis
**C11**	Harvard	C	70	Female	17.2	White	No Dementia	N/A	0	1	Arteriosclerosis, mild autolysis
**C12**	Harvard	C	76	Female	8.1	Black/African-American	No Dementia	N/A	0	1	Remote probable tentorial notching of ventral uncus, arteriosclerosis, arterial intimal hyperplasia

**C13**	Harvard	C	71	Female	14.1	Unknown	No Dementia	N/A	0	1	None reported
**C14**	Harvard	C	85	Male	20.8	White	No Dementia	N/A	0	2	Atherosclerosis, arteriosclerosis, remote microhaemorrhage in substantia nigra
**C15**	Harvard	C	77	Male	14.6	Unknown	No Dementia	N/A	0	2	Atherosclerosis, arteriosclerosis
**C16**	Harvard	C	85	Male	29.1	Unknown	No Dementia	N/A	0	1	Atherosclerosis, arteriosclerosis, mild autolysis
**C17**	Harvard	C	78	Female	22.7	Unknown	No Dementia	N/A	0	1	CVD, atherosclerosis, arteriosclerosis, remote small infarcts
**C18**	Harvard	C	66	Male	22.1	White	No Dementia	N/A	0	1	Mild arteriosclerosis, mild to moderate autolysis
**C19**	Harvard	C	84	Male	28.8	White	No Dementia	N/A	0	1	Amyloid angiopathy, atherosclerosis, arteriosclerosis, mild Purkinje cell loss, mild autolysis
**C20**	Harvard	C	74	Male	18.6	Unknown	No Dementia	N/A	0	0	None reported
**C21**	Harvard	C	65	Male	15.8	Unknown	No Dementia	N/A	0	*0/1**	None reported
**C22**	Harvard	C	68	Male	16.1	Unknown	No Dementia	N/A	0	*0/1**	None reported
**C23**	Harvard	C	83	Male	13.0	Unknown	No Dementia	N/A	0	*0/1**	None reported
**DLB1**	Sepulveda	DLB	78	Male	10	White	DLB	*Limbic**	Unknown	*2**	None reported
**DLB2**	Sepulveda	DLB	75	Male	11.9	White	DLB/AD	*Limbic**	Unknown	4/5	Carcinoma in situ of bladder, hypertension
**DLB3**	Sepulveda	DLB	68	Male	11.9	White	DLB/AD	Diffuse /cortical	Unknown	2/3	Hallucinations, paranoid personality disorder, atherosclerotic heart disease of native coronary artery, hypertension, type II diabetes mellitus
**DLB4**	Sepulveda	DLB	80	Female	12.8	White	DLB/AD	Cortical	Unknown	2/3	Osteoporosis, pneumonia, anxiety disorder, major depressive disorder

**DLB5**	Sepulveda	DLB	71	Male	20.3	White	DLB	*Limbic**	Unknown	0	Alcohol abuse, major depressive disorder, hypertension, sleep disorder, type II diabetes mellitus
**DLB6**	Sepulveda	DLB	66	Male	17.3	White	DLB	Limbic	Unknown	0	Chronic kidney disease, diabetes mellitus, anxiety disorder, hallucinations, major depressive disorder
**DLB7**	Sepulveda	DLB	68	Female	8	White	DLB/AD	Diffuse	Unknown	5	None reported
**DLB8**	Harvard	DLB	74	Male	11.0	White	DLB/AD	Cortical	Unknown	5	Amyloid angiopathy, SVD, arteriosclerosis
**DLB9**	Harvard	DLB	71	Female	9.6	White	DLB/AD	Cortical	Unknown	4	Mild atherosclerosis, arteriosclerosis, minute capillary telangiectasia
**DLB10**	Harvard	DLB	73	Male	11.3	White	DLB	Cortical	5	2	Atherosclerosis, arteriosclerosis, single minute focus of probable fat embolism (history of hip trauma)
**DLB11**	Harvard	DLB	85	Female	8.6	White	DLB	Limbic	Unknown	5	Amyloid angiopathy, arteriosclerosis
**DLB12**	Harvard	DLB	76	Female	12.8	White	DLB/AD	Limbic	Unknown	3	SVD, arteriosclerosis, atherosclerosis, multiple microinfarcts
**DLB13**	Harvard	DLB	75	Female	22.6	White	DLB	Diffuse	Unknown	2/3	Very mild amyloid angiopathy
**DLB14**	Harvard	DLB	72	Female	18.8	White	DLB/AD	Diffuse	6	6	TDP-43 proteinopathy, CVD, mild arteriosclerosis
**DLB15**	Harvard	DLB	65	Male	19.2	White	DLB	Limbic	4	2	Atherosclerosis, mild arteriosclerosis
**DLB16**	Harvard	DLB	73	Female	10.4	White	DLB	Cortical	Unknown	4	Atherosclerosis, arteriosclerosis
**DLB17**	Harvard	DLB	77	Male	15.0	White	DLB	Cortical	5	4	Moderate to severe amyloid angiopathy, SVD, atherosclerosis, arteriosclerosis, microinfarcts
**DLB18**	Harvard	DLB	79	Male	20.6	White	DLB/AD	*Limbic**	Unknown	6	CVD, remote infarcts, cerebral amyloid angiopathy, arteriolar sclerosis, moderate to severe atherosclerosis, COVID-19 (in remission), atherosclerotic heart disease of native coronary artery, chronic kidney disease, chronic obstructive pulmonary disease,

											heart failure, hypertension, type II diabetes mellitus, unspecified atrial fibrillation
**DLB19**	Harvard	DLB	80	Male	20.0	White	DLB	Cortical	Unknown	3	Mild chronic traumatic encephalopathy, small fibrous meningioma, mild remote subdural haemorrhage with subdural membrane, atherosclerosis, arteriosclerosis, benign prostatic hyperplasia with lower urinary tract symptoms
**DLB20**	Harvard	DLB	76	Male	21.3	White	DLB	Diffuse	5	2	CVD, arteriolar sclerosis, chronic mastoiditis (left ear), hyperlipidaemia

**Putative staging based on medical reports supplied by brain bank.*

AD, Alzheimer's disease; DLB, dementia with Lewy bodies; N/A, not applicable; PMD, postmortem delay

Supplementary Table A2. Cohort region summaries

	Age at Death (y)	Sex (% Male)	PMD (h)
		**HP**	
Controls (n = 14)	73.6 (65-85)	35.7	17.6 (12.2-28.8)
Cases (n = 15)	73.1 (65-85)	43.8	13.7 (8.0-23.6)*
	**MED**		
Controls (n = 15)	72.3 (65-85)	46.7	16.3 (8.1-21.3)
Cases (n = 15)	73.1 (65-85)	43.8	13.7 (8.0-23.6)
	**MTG**		
Controls (n = 15)	72.8 (65-85)	40.0	16.9 (12.2-21.3)
Cases (n = 15)	73.1 (65-85)	43.8	13.7 (8.0-23.6)*
	**CG**		
Controls (n = 15)	73.0 (65-85)	46.7	16.2 (8.1-21.3)
Cases (n = 15)	73.1 (65-85)	43.8	13.7 (8.0-23.6)
	**PVC**		
Controls (n = 16)	73.0 (65-85)	43.8	16.3 (8.1-21.3)
Cases (n = 15)	73.1 (65-85)	43.8	13.7 (8.0-23.6)
	**PONS**		
Controls (n = 14)	71.6 (65-85)	35.7	16.9 (8.1-21.3)
Cases (n = 8)	73.9 (65-85)	55.6	14.2 (8.6-23.6)
	**MCX**		
Controls (n = 16)	73.0 (65-85)	43.8	16.3 (8.1-21.3)
Cases (n = 15)	73.1 (65-85)	43.8	13.7 (8.6-23.6)
	**PUT**		
Controls (n = 13)	71.8 (65-85)	46.2	17.0 (8.1-21.3)
Cases (n = 8)	73.1 (65-85)	43.8	13.7 (8.6-23.6)
	**SN**		
Controls (n = 18)	73.7 (65-85)	38.9	18.2 (8.1-29.1)
Cases (n = 15)	73.1 (65-85)	43.8	13.7 (8.6-23.6)*
		**CB**	
Controls (n = 16)	73.7 (66-85)	38.5	18.8 (8.1-28.8)
Cases (n = 12)	75.0 (65-85)	38.5	15.7 (8.6-23.6)

Values shown for age at death and postmortem delay (PMD) are means (range). * $p < 0.05$ as determined by Welch's t-test.

Supplementary Table A3. PDD Cohort Summary – All Regions Except SN

	Gender (% male)	Age at death (y)	PMD (h)	Brain Weight (g)
**Controls (n = 9)**	44	70 (61 – 79)	19.8 (12.5 – 25.95)	1285 (1135 – 1371)
**Cases (n = 9)**	66	73 (61 – 81)	14.6 (4.3 – 21.88)	1291 (1187 – 1520)

Table shows mean (range) age, PMD, and brain weight.

Supplementary Table A4. PDD Cohort Summary – SN Only

	Gender (% male)	Age at death (y)	PMD (h)	Brain Weight (g)
**Controls (n = 9)**	44	70 (62 – 79)	20.6 (10.8 – 30.0)	1335 (1135 – 1550)†
**Cases (n = 9)**	66	73 (61 – 81)	14.6 (4.3 – 21.9)*	1291 (1187 – 1520)

Table shows mean (range) age, PMD, and brain weight. * $p < 0.05$ between cases and controls as determined by Mann-Whitney U Test.
†Brain weight not available for one control.

Supplementary Table A5. Parkinson's disease dementia (PDD) cohort characteristics

Code	Sex	Age at death	Clinical diagnosis	α-syn Braak stage	Post-mortem Delay (h)	Whole-brain weight (g)	Cause of Death
C1	Male	61	No dementia present	0	12.5	1182	Respiratory failure; heart failure; coronary artery disease
C2	Male	71	No dementia present	0	23.9	1371	Acute myocardial infarction; severe coronary artery disease; pulmonary oedema; diabetes mellitus; cardiopulmonary arrest
C3	Male	74	No dementia present		25.5	1300	Aortic dissection
C4	Male	70	No dementia present	0	12.7	1350	Atherosclerotic and hypertensive heart disease
C5	Female	68	No dementia present	0	19.1	1270	Acute myocardial infarction; coronary artery disease
C6	Female	65	No dementia present	0	19.4	1372	Hypertensive arteriosclerotic cardiovascular disease; morbid obesity; respiratory arrest; suspected embolus
C7	Female	77	No dementia present		21.4	1135	Pending death certificate
C8	Female	79	No dementia present	0	17.8	1300	Acute myocardial infarction; coronary artery disease; atrial fibrillation; COPD
C9	Female	67	No dementia present	0	25.9	1382	Pending death certificate
PDD1	Male	61	PDD	Unknown	13.8	1188	Cardiopulmonary arrest; probable acute myocardial infarction; Parkinson's disease
PDD2	Male	79	PDD	Unknown	16.2	1250	End stage Parkinson's disease
PDD3	Male	71	PDD	III-IV	16.2	1262	Pending death certificate
PDD4	Male	78	PDD	VI	20.4	1520	Respiratory failure; aspiration pneumonia; dysphagia; Parkinson's disease
PDD5	Male	70	PDD	Unknown	4.3	1218	Aspiration pneumonia; Parkinson's disease
PDD6	Female	69	PDD	V	17.5	1187	Pending death certificate
PDD7	Female	81	PDD	Unknown	7.0	1415	End stage Parkinson's disease
PDD8	Female	79	PDD	Unknown	21.9	1200	Respiratory failure; Parkinson's disease
PDD9	Female	67	PDD	Unknown	14.5		Lewy body disease; Parkinson's disease

Supplementary Table A6. AD cohort summary

	Gender (% male)	Age at death (y)	PMD (h)	Brain Weight (g)
**Controls (n = 9)**	53.8	73 (61-78)	12 (5.5-15.0)	1260 (1094 – 1461)
**Cases (n = 9)**	55.6	72 (60-80)	7 (4.0-12.0)	1062 (831 – 1355)

Table shows mean (range) age, PMD, and brain weight.

Supplementary Table A7. AD cohort characteristics

Code	Gender	Age at death	Clinical diagnosis	Braak stage (AD)	Post-mortem delay (h)	Whole-brain weight (g)	Brain pH	Cause of Death
AD1	M	60	Alzheimer's Disease	VI	7.0	1020	7.0	Alzheimer’s disease
AD2	F	62	Alzheimer's disease	VI	6.0	831	6.0	Alzheimer’s disease
AD3	F	63	Alzheimer's disease	VI	7.0	1080	7.0	Bronchopneumonia
AD4	F	70	Alzheimer's disease	V	7.0	1044	7.0	Lung cancer
AD5	M	73	Alzheimer's disease	IV	4.0	1287	4.0	GI hemorrhage
AD6	F	74	Alzheimer's disease	V	8.5	1062	8.5	Metastatic cancer
AD7	M	74	Alzheimer's disease	VI	12.0	1355	12.0	Pseudomonas bacteremia
AD8	M	77	Alzheimer's disease	VI	4.5	1180	4.5	Myocardial infarction
AD9	M	80	Alzheimer's disease	V	5.5	1039	5.5	Bronchopneumonia/ pulmonary oedema
C1	M	61	No dementia or brain disease	0	7.0	1258	7.0	Ischemic heart disease
C2	F	64	No dementia or brain disease	0	5.5	1260	5.5	Pulmonary embolism
C3	F	63	No dementia or brain disease	0	12.0	1280	12.0	Ruptured aorta
C4	F	72	No dementia or brain disease	0	9.0	1230	9.0	Emphysema
C5	M	66	No dementia or brain disease	0	9.0	1461	9.0	Ischemic heart disease
C6	F	76	No dementia or brain disease	II	12.0	1094	12.0	Metastatic carcinoma
C7	M	73	No dementia or brain disease	0	13.0	1315	13.0	Ischemic heart disease
C8	M	78	No dementia or brain disease	0	7.5	1260	7.5	Ruptured aortic aneurysm
C9	M	78	No dementia or brain disease	0	12.0	1416	12.0	Ruptured MI

Supplementary Table A8. HD cohort summary

	Gender (% male)	Age at death (y)	PMD (h)	Brain Weight (g)
**Controls (n = 9)**	66.6	67 (49-81)	10.6 (6.5-15)	1322 (1210-1495)
**Cases (n = 9)**	66.6	65 (51-80)	11.2 (7-15)	1104 (787-1497)

Supplementary Table A9. HD cohort characteristics

Sample	Status	Age (y)	Sex	PMD (h)	Brain weight (g)	CAG repeats	CAP Score	Vonsattel Grade	Cause of Death
**H01**	HD	80	M	9	1105	20/40	123.3	1	Bronchopneumonia
**H02**	C	63	M	9	1432	19/20	-	-	Ischemic Heart Disease
**H03**	HD	67	F	9	1139	15/42	123.9	1	Myocardial Infarction
**H04**	C	61	M	7	1258	17/19	-	-	Ischemic Heart Disease
**H05**	C	77	F	12	1227	18/20	-	-	Coronary Atherosclerosis
**H06**	HD	59	F	7	787	23/47	154.5	4	Bronchopneumonia
**H07**	C	73	F	13	1210	17/17	-	-	Ischemic Heart Disease
**H08**	HD	62	F	11	826	17/45	143.3	3	Bronchopneumonia
**H09**	C	73	M	13	1315	17/23	-	-	Ischemic Heart Disease
**H10**	HD	62	M	9	992	18/43	124.2	2	Huntington's disease
**H11**	C	49	M	13	1495	17/17	-	-	Ischemic Heart Disease
**H12**	HD	83	M	13	1168	17/42	153.5	1	Bronchopneumonia
**H13**	C	66	M	15	1360	15/20	-	-	Ischemic Heart Disease
**H14**	HD	58	M	14	1497	28/44	125.1	2	Bronchopneumonia
**H15**	C	64	F	6.5	1260	18/23	-	-	Pulmonary Embolism
**H16**	HD	51	M	15	1200	10/46	125.7	2	Bronchopneumonia
**H17**	C	81	M	7	1343	15/18	-	-	Coronary Atherosclerosis

All HD samples were confirmed to have the disease with genetic testing, with all having >40 CAG repeats. The most common cause of death in control samples was ischemic heart disease, whereas bronchopneumonia was the most common cause in HD donors.

Supplementary Figure A1. Pantothenic acid alterations in rat cortex and cerebellum with increasing PMD

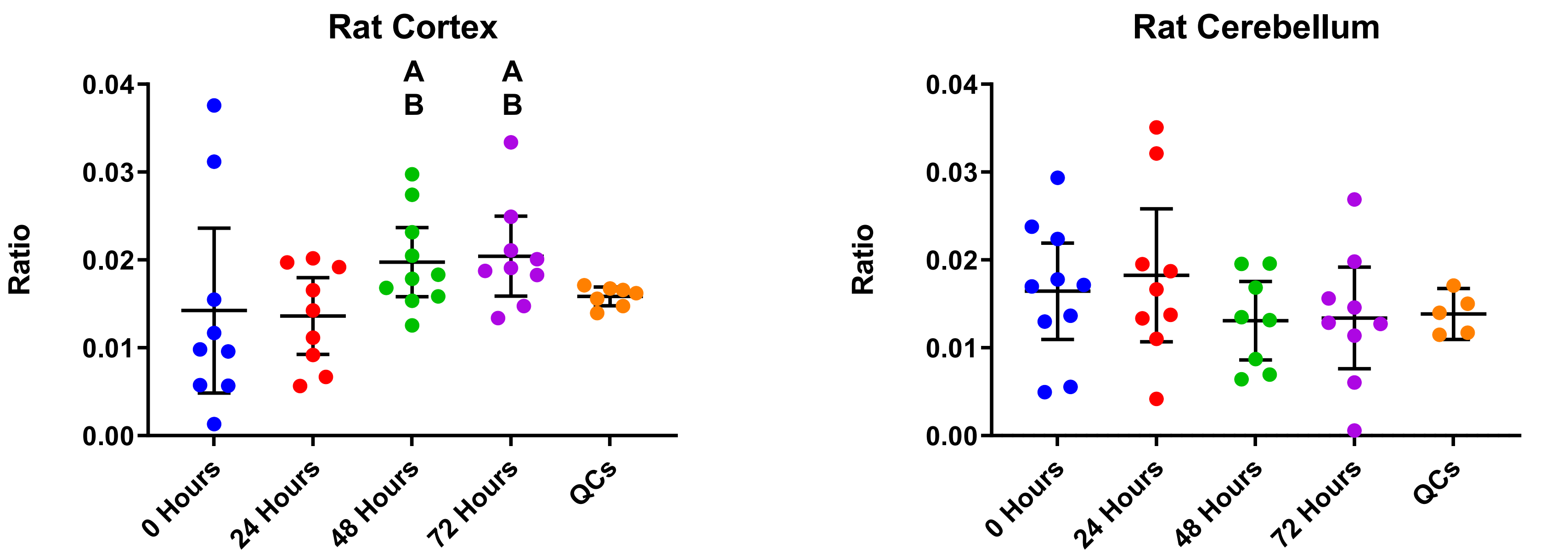

In a previous study [1], the effects of PMD on several metabolites including pantothenic acid were determined in two regions of the rat brain, from 0 h PMD to 72 h. This analysis was performed in rat brain in order to avoid confounding by the many factors that may differ between human samples (e.g., age, sex, health conditions, etc.). The figure shows pantothenic acid levels, displayed as ratio to internal standard. **A** indicates significant difference to 0 h PMD; **B** indicates significant difference to 24 h PMD. QC, Quality control.

[1] Scholefield M, Church SJ, Xu J, Robinson AC, Gardiner NJ, Roncaroli F, Hooper NM, Unwin RD, Cooper GJS (2020) Effects of alterations of postmortem delay and other tissue-collection variables on metabolite levels in human and rat brain. *Metabolites* **10**, 438.

Supplementary Table A10. Power analysis

Region	Statistical Power ($p < 0.05$)	Statistical Power ($p < 0.01$)	Minimum N Required Per Group ($p < 0.05$)	Minimum N Required Per Group ($p < 0.01$)
Cerebellum	52.4%	28.9%	33	48
Cingulate Gyrus	56.2%	32.3%	40	60
Motor Cortex	89.9%	74.6%	18	27
Medulla	32.2%	14.0%	62	92
Pons	98.9%	95.2%	11	16
Substantia Nigra	94.6%	84.0%	12	18
Hippocampus	99.3%	96.6%	7	11
Putamen	58.2%	34.2%	36	54
Middle Temporal Gyrus	62.2%	38.0%	27	40
Primary Visual Cortex	89.9%	74.6%	16	27

Table shows statistical power at $p < 0.05$ and $p < 0.01$, shown in %. A power of ≥80% was considered good. Table also shows minimum N numbers per group required to confidently determine case–control differences at $p < 0.05$ and $p < 0.01$.
